# Real-to-virtual domain transfer-based depth estimation for real-time 3D annotation in transnasal surgery: a study of annotation accuracy and stability

**DOI:** 10.1007/s11548-021-02346-9

**Published:** 2021-03-30

**Authors:** Hon-Sing Tong, Yui-Lun Ng, Zhiyu Liu, Justin D. L. Ho, Po-Ling Chan, Jason Y. K. Chan, Ka-Wai Kwok

**Affiliations:** 1 Department of Mechanical Engineering, The University of Hong Kong, Pokfulam Road, Hong Kong; 2 Department of Otorhinolaryngology, Head and Neck Surgery, The Chinese University of Hong Kong, Sha Tin, Hong Kong SAR

**Keywords:** Augmented reality, Surgical annotation, Monocular depth estimation, Domain transfer learning, Transnasal surgery

## Abstract

**Purpose:**

Surgical annotation promotes effective communication between medical personnel during surgical procedures. However, existing approaches to 2D annotations are mostly static with respect to a display. In this work, we propose a method to achieve 3D annotations that anchor rigidly and stably to target structures upon camera movement in a transnasal endoscopic surgery setting.

**Methods:**

This is accomplished through intra-operative endoscope tracking and monocular depth estimation. A virtual endoscopic environment is utilized to train a supervised depth estimation network. An adversarial network transfers the style from the real endoscopic view to a synthetic-like view for input into the depth estimation network, wherein framewise depth can be obtained in real time.

**Results:**

(1) Accuracy: Framewise depth was predicted from images captured from within a nasal airway phantom and compared with ground truth, achieving a SSIM value of 0.8310 ± 0.0655. (2) Stability: mean absolute error (MAE) between reference and predicted depth of a target point was 1.1330 ± 0.9957 mm.

**Conclusion:**

Both the accuracy and stability evaluations demonstrated the feasibility and practicality of our proposed method for achieving 3D annotations.

## Introduction

Any surgical procedure involves the collaboration between different personnel like surgeons and nurses. Effective communication is paramount to ensuring a smooth surgical workflow. In particular, communication can be achieved by graphical annotations drawn on a display device when the use of an endoscope is involved. In this manner, any target structures inside the field of view can be annotated with information and instantly shared among related personnel. Advantages brought by surgical annotation are not limited to within an operation theatre. As it enables real-time graphical communication, beneficiaries include everyone involved in the procedure such as teachers, students and medical trainees. Examples of annotation include a multi-institutional cooperation during adrenalectomy through video conferencing [1], and an experimental illustration of intention sharing by visualizing eye gazes of separated collaborators [2]. Both examples involved graphical annotations to facilitate effective communication. Nevertheless, these approaches only applied 2D annotations on static endoscopic views. Annotations failed to anchor rigidly with respect to the patient anatomy upon camera movement [3].

In this work, we aim to achieve 3D annotation in which annotations made in an endoscopic view would anchor in a stable and accurate way to the target surface during camera movement with the aid of endoscope pose tracking by an EM sensor and monocular depth estimation. Not only do annotations anchor to the surgical scene during camera movement, size change with respect to endoscopic view as the camera approaches the annotated target may provide viewers with improved depth perception. To elaborate, achieving 3D annotation is essentially implementing augmented reality (AR). The 3D annotation is instantiated in a virtual 3D world and later registered to the real-world surgical field. By augmenting the exposed surgical view with intra- or pre-operatively obtained images or 3D models [4], AR applied in surgeries allow overlay of subsurface critical structures and pre-operatively planned trajectories that include depth information. Subsequently, it may reduce the risk of complications, increase surgical efficiency and aid with surgical training [5].

As a proof of concept, we selected nasal surgery for the implementation of 3D annotation. AR systems are the most useful when the target surgical sites have little deformation and movement [6], making the nasal cavity and paranasal sinuses a suitable candidate for AR implementation. Additionally, due to its proximity to the brain, many critical structures can be overlaid in the endoscopic view. To achieve AR in nasal surgeries, researchers and companies tend towards sensor-based approaches utilizing external equipment. The endoscope and the target anatomy are usually tracked by medical graded optical or EM trackers. The Scopis$\circledR$ Hybrid Navigation is a commercial example that combines optical and EM sensing to achieve AR. Next, pre-operative (pre-op) 3D models are usually obtained from computed tomography (CT) scans, which is then registered to the sensor-based tracking system reference frame by rigid registration, enabling overlay of pre-operatively obtained models onto the real anatomy in the surgical scene.

Provided that we adopt the above approach to achieve 3D annotation, depth information observed by the camera would be based on the registered pre-op 3D model. However, observed depth in this context may not be representative of the real surface during surgery, especially in the nasal cavity where soft mucosal linings are not clearly observable in CT scans. It is also notable that the quality of a 3D reconstruction from CT scans is highly dependent on scanning quality, reconstruction software and human operation [6].

One of the possible alternatives to obtain depth is to resort to traditional vision-based approaches such as stereo or monocular visual Simultaneous Localization and Mapping (vSLAM). vSLAM outputs camera trajectory and a 3D structure of an environment without any prior knowledge of the environment or the use of any active sensors. Using vSLAM, visual input can be taken advantage of to perform tracking and mapping. Depth can be obtained from vision in real-time, which may be more representative than depth based on a registered pre-op model. Real-time stereo reconstruction has been performed previously for laparoscopic surgery [7]; however, stereo vision is difficult to implement in nasal surgeries due to constraints on the endoscope size. Depth estimation through monocular endoscopes has also been demonstrated, for example, by tracking and matching video frame feature points for both endoscope tracking and point cloud reconstruction in the nasal airway of a cadaver head [8] and through ORBSLAM [9] approaches for tracking laparoscope pose and mapping the surgical scene [10]. However, feature-based tracking is prone to failure inside the nasal cavity owing to the lack of texture and the apparent repetition of patterns [11].

**Fig. 1:**
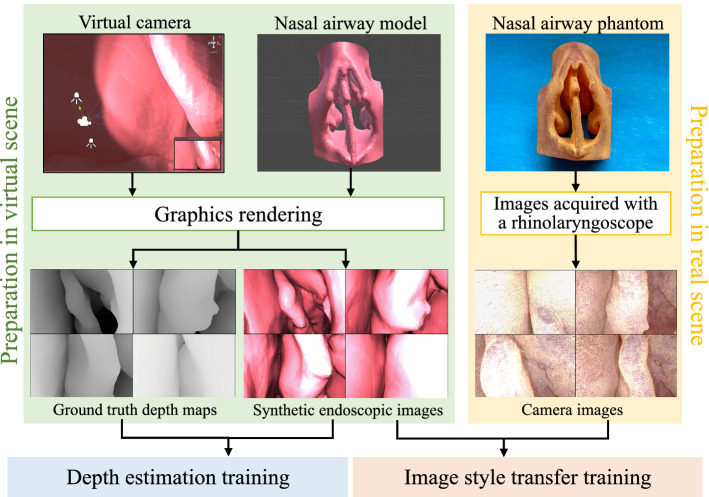
Overview of preparation process for ground truth depth maps, synthetic endoscopic images and real endoscopic images for depth estimation and image style transfer training

In view of the rapid development in deep learning-based monocular depth estimation, there lies a great opportunity in surgical AR to exploit vision-based depth. Novel examples giving promising estimation results include DORN [12] and DenseDepth [13]. Supervised learning methods such as DORN require an endoscopic image dataset with ground truth depth labels for training. During the application phase of the trained neural network, the estimated depth output is generated from colour image input. Unfortunately, there does not exist a large, readily available labelled dataset for the nasal airway. It is also impractical to collect ground truth depth data inside the nasal airway using active sensors. To address this limitation, some researchers [14, 15] have attempted to train their depth estimation networks in a self-supervised manner such that no depth labelling is required prior to network training. Nonetheless, both employed a structure from motion (SfM) algorithm to obtain sparse depth before the training phase. Consequently, depth estimation output highly depends on the accuracy and quality of SfM output. Implicit domain adaptation that translates synthetic colon endoscopic images to depth maps by using pix2pix [16] has also recently been proposed. Other than paired simulated data, unlabelled real colon images were also involved in the training phase such that the trained model may produce more accurate depth predictions in patient data [17].

Adopting a similar approach used in prior art [18], we train a supervised depth estimation network in a virtual environment and utilize it to predict depth of real endoscopic image. Prior to depth estimation, a real-to-virtual image style transfer using cycle generative adversarial network (cycleGAN) [19] is performed. With adversarial learning, domain adaptation between the real domain and the synthetic domain is accomplished. Previously, cycleGAN-like architectures has been used to adapt real bronchoscopy images to virtual style images [20]. Real-to-virtual adaptation was also used for colonoscopy using a generative adversarial network (GAN) architecture [21]. Through this approach, preparation of an unlimited amount of absolute ground truth depth becomes possible, while depth prediction can be implemented on real-to-virtual-adapted real endoscopic images. Time and labour cost for data preparation through this approach would be minimal. In addition, real-to-virtual domain adaptation can remove patient-specific texture details that may vary widely between patients, potentially making the depth estimation network generalizable across patients [21].

Apart from aiming at generating depth that is more representative of a surface so as to increase 3D annotation accuracy, we are also concerned with its stability. Therefore, a brief stability evaluation of our proposed system is included towards to end of this study. Additionally, datasets used in training and testing are provided for research purposes.^1^ Major contributions of this study are listed below:

1. The application of monocular depth estimation is extended beyond offline 3D reconstruction of surgical scenes, into applications with real-time augmented reality for surgical guidance.
2. A supervised depth estimation network is trained entirely in a virtual environment and used to predict depth from endoscopic images in real-time by implementing cycleGAN-based real-to-virtual style transfer.
3. Predicted depth is quantitatively evaluated against ground truth depth in a nasal airway phantom. Accuracy of augmented 3D annotations are evaluated, while overall system stability is quantitatively assessed.

**Fig. 2:**
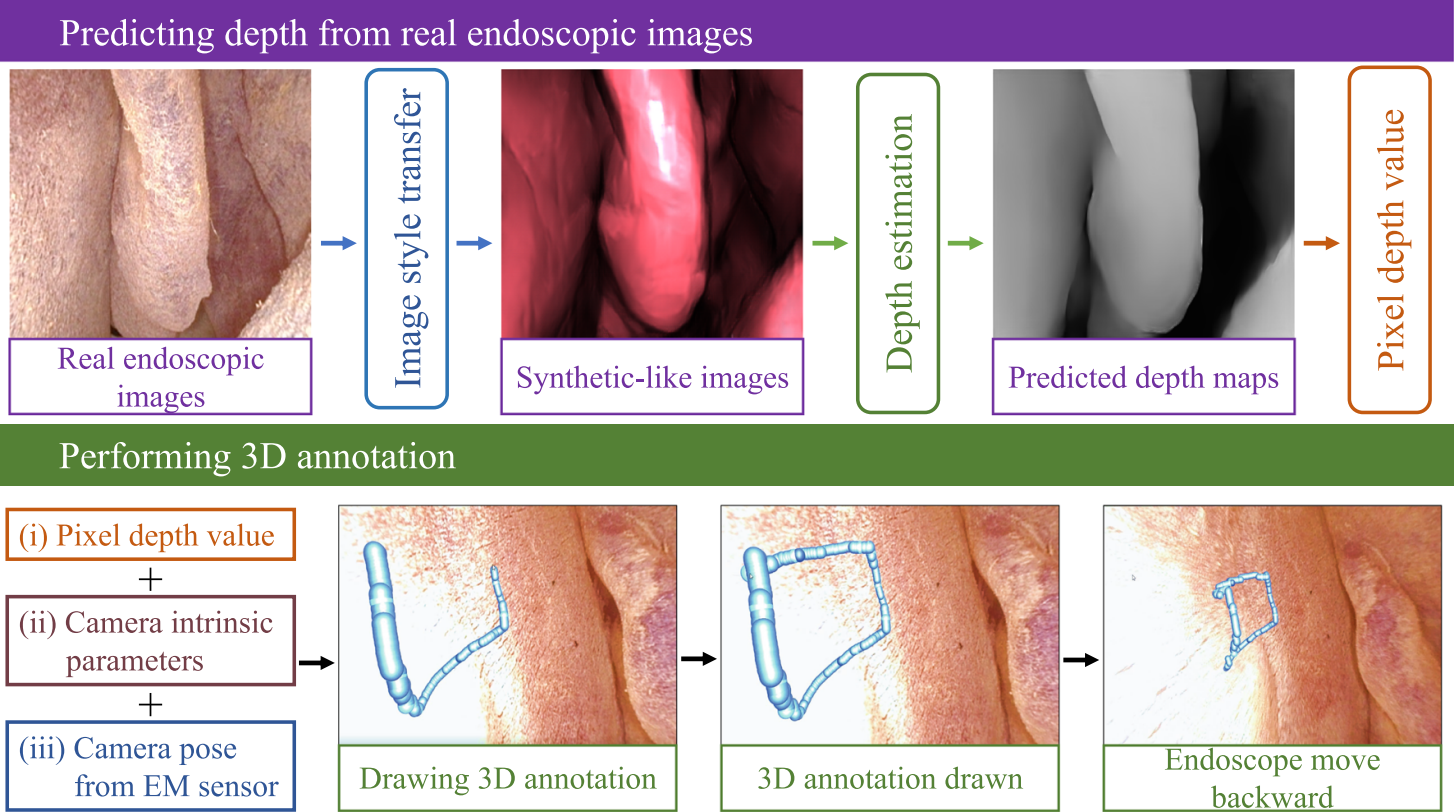
Integration of image style transfer and depth estimation networks to achieve real-time 3D annotation. Based on (i) predicted depth, (ii) camera intrinsic parameters and (iii) camera pose from EM sensor, annotations can be anchored to the anatomical surface in a stable manner

## Methodology

### Data preparation for deep neural network (DNN) training

The goal of our method is to train a supervised depth estimation network in a virtual environment and utilize it to predict real endoscopic image depth. In doing so, synthetic endoscopic images and the corresponding ground truth depth maps were generated in a virtual world space using Unity3D. An overview of the data preparation process is illustrated in Fig. 1. A virtual camera was set up with intrinsic parameters obtained from camera calibration of an Olympus rhinolaryngoscope (ENF-VH). Not only did we match intrinsic parameters of the real and virtual endoscopes, but we also attached two point light sources near the virtual camera that exhibit realistic inverse square intensity fall-off in relation to distance from the source. Next, an anatomically accurate nasal airway model was imported into the virtual environment. The model was obtained from a CT scan of a cadaver head, followed by 3D segmentation and model editing. The model surface was assigned a uniform light-red colour to emulate the nasal mucosal lining, however, noting that patient-specific textures such as vascular patterns were absent. Depth estimation network trained in this manner is hypothesized to have improved generalizability across patients.

A dataset of 3600 synthetic endoscopic images and the corresponding ground truth depth maps were captured from the virtual camera while being moved inside the virtual nasal airway model along a pre-defined pathway. Ground truth depth maps were stored as greyscale images. Depth observed by the virtual camera was set to span a range of 0.01–25 mm. As we adopted a GAN-based unpaired image-to-image style transfer network, real endoscopic images and synthetic endoscopic images prepared do not necessarily correspond with one another. Therefore, 3000 real endoscopic images were directly captured inside a 3D-printed nasal airway phantom. The phantom was based on a segmented anatomically accurate nasal airway model and 3D-printed in a material with shore hardness value of 70.

### Real-to-virtual image style transfer

Our aim for real-to-virtual image style transfer is to learn a mapping $G:X\rightarrow Y$, where the domain variance between real RGB image $x \in X$ and synthetic-style RGB image $y \in Y$ is bridged. As a result, a depth estimation network trained on synthetic endoscopic images can be deployed on real RGB endoscopic images. To obtain the mapping model, a GAN-based unpaired image-to-image translation method called cycleGAN [19] is applied. It consists of two translators *G* , *F* to learn the mapping functions $G:X\rightarrow Y$ and $F:Y\rightarrow X$. Two adversarial discriminators $D_x$ and $D_y$ are trained to differentiate the style-transferred images from the domain images. The goal of discriminator $D_x$ is to distinguish $\{x\}$ and $F\{(y)\}$ which is style-transferred to style of *X* by translator *F* and vice versa for $D_y$ and *G*. $D_x$ and $D_y$ are both PatchGAN [16] classifiers. Transformer *G* is thus encouraged to translate *X* into outputs indistinguishable from domain *Y* . In other words, *G* is enforced by $D_y$ to produce synthetic-like images from real RGB images. The loss function combines adversarial loss [22] and cycle consistent loss [19].

### Depth estimation

The architecture of our depth estimation model is an encoder–decoder network DenseDepth presented in [13]. The encoder part is a pretrained DenseNet-161 network for extracting features from our RGB images and representing them as a feature map. The decoder part contains blocks of convolutional and up-sampling layers to transform the feature map into the desired depth output. The same spatial shape of the encoder layers is skip-connected into the decoder to improve the prediction performance and produce sharper depth estimations. The loss function to train our network is a combination of depth loss and structural similarity (SSIM) loss. The aim of the model is to learn the mapping between the synthetic endoscopic image dataset and the corresponding ground truth depth value such that it can accurately predict the depth value in real endoscopic images.

### System integration in the virtual scene to achieve 3D annotation

After the training phase of both depth estimation and image style transfer networks, system integration was performed to achieve 3D annotation using Unity3D as an interface for visualization (Fig. 2). First, a six-degree-of-freedom (DoF) electromagnetic (EM) sensor (Aurora, NDI Medical, Canada) was attached to the tip of the endoscope. Using Tsai’s method [23], hand–eye calibration was employed to find the transformation $\mathbf {^cT_s}$, a description of the sensor frame relative to the camera frame. To reduce error propagation in the case of imperfect hand–eye calibration, the EM sensor was attached to the endoscope tip at approximately 2 mm from the camera’s optical axis. By directly streaming sensor pose in EM frame $\mathbf {^{em}T_s}$ into Unity3D, pose of the camera tip with respect to virtual world $\mathbf {^wT_c}$ was assigned as the virtual camera pose:

1 $$\begin{aligned} \mathbf {^wT_c} = \mathbf {^{em}T_s} \cdot \mathbf {^cT_s}^{-1} \end{aligned}$$

Next, RGB video frames were streamed from the endoscope during observation of the 3D-printed nasal airway phantom, which was static relative to the EM tracking field. Before passing video frames to i) Unity3D for visualization and ii) real-to-virtual image style transfer network, image undistortion was applied as a data pre-processing step. Undistorted frames passed to (i) may then accurately be overlaid with any virtual object, which would be observed by a virtual camera that was distortion-free by default.

Style-transferred image frames processed in (ii) were further relayed to the depth estimation network for generating framewise depth maps. Depth at each pixel was stored as a normalized float number in a range between 0 (far) and 1 (near), which was then converted into a depth range of 0.01–25 mm in the virtual environment, matching the depth range of the image set used for depth estimation training.

While RGB frames were displayed in the Unity3D view in real time, a pixel (*u*, *v*) was selected by the cursor to begin annotation. Given the camera intrinsic matrix ${\mathbf {K}}$ and depth value *d* at (*u*, *v*) retrieved from a predicted depth map, an annotation element in the form of a simple spherical object with a position $\mathbf {p_c = }$ $$\begin{bmatrix} x_c&y_c&z_c \end{bmatrix}\mathrm {^T}$$ in camera coordinates was placed in virtual game world, where

2 $$\begin{aligned} \mathbf {p_c} = \begin{bmatrix} x_c \\ y_c \\ z_c \end{bmatrix} ={\mathbf {K}}^{-1} \cdot d \cdot \begin{bmatrix} u \\ v \\ 1 \end{bmatrix} \end{aligned}$$

which was further expressed as $\mathbf {p_w}$ in virtual world coordinates:

3 $$\begin{aligned} \begin{bmatrix} \mathbf {p_w} \\ 1 \end{bmatrix} = \begin{bmatrix} x_w \\ y_w \\ z_w \\ 1 \end{bmatrix} = \mathbf {^wT_c} \cdot \begin{bmatrix} \mathbf {p_c} \\ 1 \end{bmatrix} =\mathbf {^{em}T_s} \cdot \mathbf {^cT_s}^{-1} \cdot \begin{bmatrix} \mathbf {p_c} \\ 1 \end{bmatrix} \end{aligned}$$

## Experiments

### Implementation of DNNs

*Image style transfer training* Before the training phase, real endoscopic images and synthetic endoscopic images prepared with the pipeline described in “Data preparation for deep neural network (DNN) training” section were resized to 288$\times$256 pixels. The translators consisted of 2 convolution layers with stride of 0.5, 9 residual blocks [24] and another convolution layer that outputs a feature map. For the discriminators, 70$\times$70 PatchGANs [16] were employed. The entire network was trained for 200 epochs with a batch size of 1. Adam [25] optimizer with initial learning rate of 0.0002 was applied. Weight parameter $\lambda$ in [19] was set to be 10.

*Depth estimation training*  The depth network was first trained with NYU Depth v2 [26] dataset as a pretraining step to obtain optimal layer weights for depth estimation. The network was trained with Adam [25] optimizer, initial learning rate 0.0001 and batch size of 4 for 20 epochs. To train the network for our purpose, synthetic endoscopic images and the corresponding depth maps were used as a subsequent fine-tune training with 50 epochs. Images were resized to 640$\times$480 pixels prior to fine-tune training. Weight parameter $\lambda$ in [13] was set to be 0.1. The proposed framework was implemented on a computer with an AMD Ryzen Threadripper 3960X CPU, 64GB RAM and two NVIDIA GTX 1080Ti GPU.

**Fig. 3:**
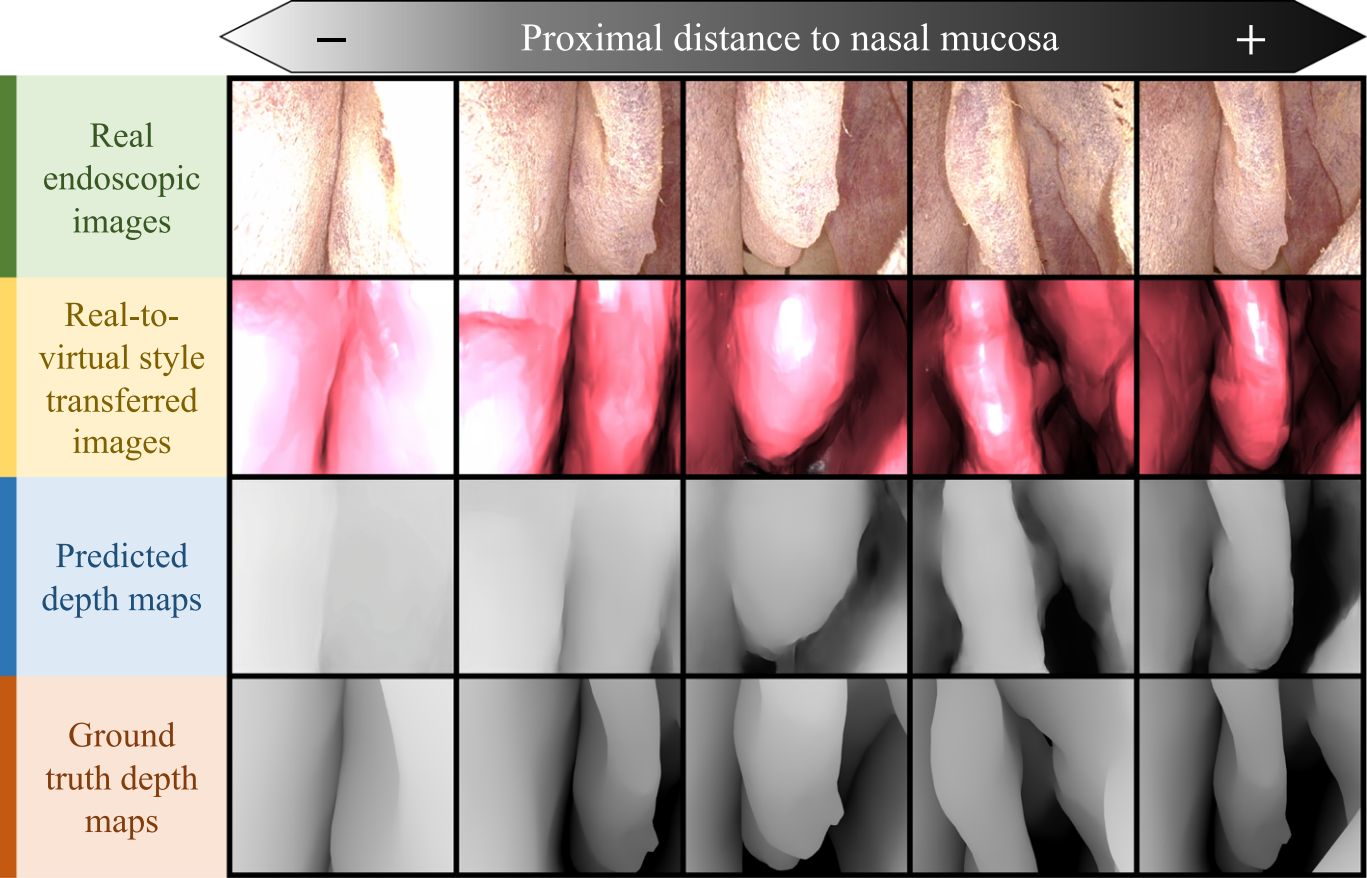
Qualitative results of predicted depth with comparison to ground truth depth

### Evaluation dataset preparation

To evaluate depth estimation accuracy, a testing dataset consisting of 2400 RGB image frames captured by the endoscope during observation of the 3D-printed nasal airway phantom was prepared. Simultaneously, corresponding ground truth depth maps were generated in the following manner:

1. By equation (1), the endoscope pose in the EM frame was assigned as the virtual camera pose in real time.
2. Having the anatomically accurate nasal airway model, six non-co-planar anatomical positions ${\{x_i\}}$ on the model were recorded in model frame.
3. With a 6-DoF EM probe, the corresponding six anatomical positions ${\{y_i\}}$ on the 3D-printed nasal airway phantom statically placed in EM tracking field were recorded.
4. To find the transformation matrix $\mathbf {^{em}T_m}$ that registers the nasal airway model to the 3D-printed phantom, where $$\mathbf {^{em}T_m} = \big ({\begin{matrix} &{} {\mathbf {R}} &{} &{} {\mathbf {t}} \\ 0 &{} 0 &{} 0 &{} 1 \end{matrix}}\big )$$, for ${\mathbf {R}}$ being the rotational matrix and ${\mathbf {t}}$ being the translational vector, ${\mathbf {R}}$ and ${\mathbf {t}}$ were solved by minimizing the least-squares error $\sum _{i=1}^{N} || {\mathbf {R}}x_i + {\mathbf {t}} - y_i ||^2$, which in our case $N = 6$, solved using singular value decomposition (SVD) method proposed in [27].
5. When importing the nasal airway model into the virtual scene, $\mathbf {^{em}T_m}$ was applied to it. As both the phantom and endoscope were registered to the virtual world, ground truth depth maps could be collected, while real RGB frames were being captured.

### Annotation stability evaluation

Stability of an AR system can be described by the synchronization of virtual object and real object movement on a display during camera motion. A measure to maximize stability is to minimize latency discrepancy between all live data streams, namely i) EM sensor pose, ii) endoscopic video stream and iii) depth predicted. While (iii) has a higher latency than (i) and (ii), synchronization can be achieved by manually adding delay on streams (i) and (ii) accordingly.

Another way to describe stability is consistency in depth predicted. Prediction made by supervised monocular depth estimation often flickers due to independent per-frame processing [28]. In our system, although depth is only assigned to a sphere annotation when the curser is clicked at a pixel, depth consistency between frames is still relevant when depth is continuously read, and sphere annotations are consecutively made as the pressed cursor is dragged.

Through the registration method described in “Evaluation dataset preparation” section, the nasal airway model was registered to the phantom statically placed in the EM tracking volume. To evaluate consistency, a virtual sphere was placed on the airway wall at a point with absolute location known. The endoscope was then directed at this sphere and moved in a forward–backward direction such that depth from sphere to camera varies with time. This depth value can be directly obtained in the virtual world as this is the distance between virtual camera and virtual sphere, which we define as the “reference depth”. Simultaneously, pixel coordinates of this sphere appearing in the Unity3D viewport was continuously captured and relayed to the depth estimation network. The corresponding predicted depth was obtained, which we define as the “predicted depth”. The reference depth and predicted depth were then captured and plotted against time. In total, five trials were conducted, each lasting for 30-60 seconds. The endoscope speed was kept below 3 mm/s, and the data sampling rate was 50 Hz.

**Table 1:** Depth prediction results: comparison between our method and state-of-the-art

Method	NRMSE	SSIM
DiL [30]	0.50	0.32
Mahmood et al. [21]	0.23	0.77
Our method	0.3224 ± 0.0773	0.8310 ± 0.0655

**Fig. 4:**
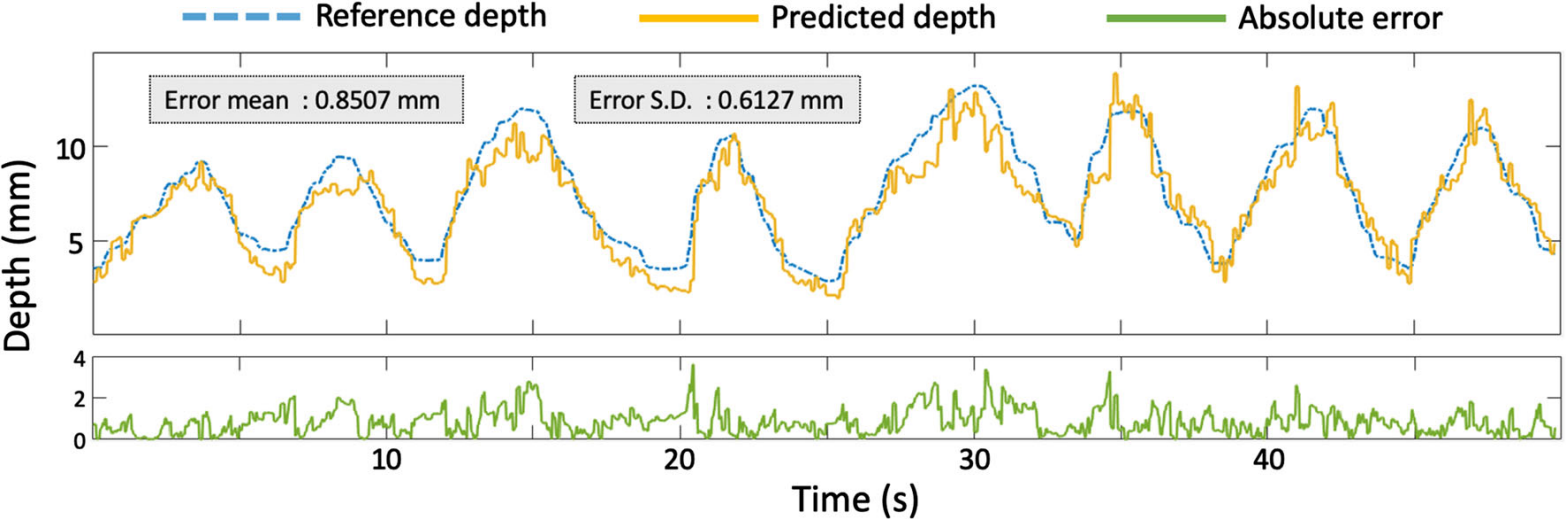
Plot depicting one trial of the depth consistency evaluation. Reference depth and predicted depth were captured during forwards-backwards movement of the endoscope in the nasal phantom airway. The endoscope speed was kept below 3 mm/s, and sampling rate was 50 Hz

## Result and discussion

### Result of depth estimation and 3D annotation implementation

Qualitative comparison between predicted depth and corresponding ground truth depth maps collected with the method described in “Implementation of DNNs” section is shown in Fig. 3. In order to quantify our depth estimation accuracy by comparing ground truth depth and predicted depth, normalized root mean square error (NRMSE) $\sqrt{\frac{\sum _{i}(x_i-y_i)^2}{n}}(x_{max}-x_{min})^{-1}$ and SSIM proposed in [29] were calculated. Similarity is indicated by a NRMSE close to 0 and a SSIM close to 1, where SSIM spans between -1 and 1. Depth prediction accuracy is shown in Table 1, which is juxtaposed to quoted prediction results of two existing methods that adopted a similar depth estimation workflow for colonoscopy [21] and that used dictionary learning (DiL) trained on CT colonoscopy images [30]. To illustrate real-time capability of our method, a supplementary video^2^ showing 3D annotation implementation can be referred to.

As compared to state-of-the-art shown in Table 1, Mahmood et al. [21] and our proposed method was significantly more accurate than the DiL implementation [30]. This could be attributed to the fact that their work did not incorporate a virtual camera model with point light sources exhibiting realistic inverse square intensity fall-off, which is believed to be a crucial element to consider in depth estimation. Despite achieving the best estimation accuracy in terms of SSIM, Fig. 3 illustrates that our method will produce poorer depth estimation results when the endoscope is closer to the nasal mucosa. This is in part likely due to light intensity saturation when moving the endoscope closer to a surface, where edges and depth information of narrow passages tend to be lost, yielding an average depth biased towards a high proximity value. We believe that there is still room for improvement such that the predicted depth can be applied to not only performing 3D annotation, but also robotic control that demands higher depth accuracy and precision. Future work may include a thorough endoscope photometric calibration to further match light properties of the virtual camera light with that of the endoscope.

### Quantitative result of system stability

Stability in terms of geometric consistency was evaluated with the method described in “Annotation stability evaluation” section. Average mean absolute error (MAE) between reference depth and predicted depth of all five trials was 1.1330 ± 0.9957 mm (or 5.5%-8.5% of the full 25 mm observable depth range of the virtual camera). While indicating a high accuracy, a low precision is revealed by a standard deviation being comparable to the MAE. The results showed rapid fluctuation of the predicted depth due to independent per-frame processing as described in Luo et al [28]. This kind of geometric inconsistency in a temporal context can also be observed in Fig. 4, which depicts the entire data logging process of one of the five trials. The predicted depth exhibited drastically more fluctuations than the reference depth. A possible remedial measure is to carry out a coupled estimation of both camera pose and depth, which is one of our aims in future work.

Although fluctuation in the predicted depth exists, the scale of predicted depth matches relatively well with the reference depth as shown in Fig. 4. A possible explanation is that the travel distance of the endoscope inside the nasal airway is relatively small compared to other anatomical sites like the colon, where scale drift is often observed in monocular depth estimation. In addition, our method design has indirectly minimized scale drift of the predicted depth. The depth prediction network we employed has a deterministic mapping which would likely output inaccurate results when inputs deviate from training data to a large extent. However, images are style-transferred to virtual-like images before being inputted into the depth estimation network. As long as virtual-like images resemble synthetic endoscopic images, predicted depth maps should be fairly coherent and consistent with respect to geometric scale.

## Conclusion

In this work, we proposed a method to achieve real-time 3D annotation in a transnasal setting. Framewise depth is predicted from real-to-virtual domain transferred endoscopic images captured from within a nasal airway phantom, achieving a SSIM value of 0.8310 ± 0.0655. 3D annotation was achieved by integrating the EM-tracked endoscope pose with real-time predicted depth based on camera frames. Both the accuracy and stability evaluations demonstrated the feasibility and practicality of our proposed method. Although our current work involves only a phantom for evaluation, we believe this preliminary work provides a capable proof of concept for future development towards a more generalizable system. By creating cadaver and patient nasal airway video datasets alongside CT images for generation of virtual models, future work will focus on proving system generalizability across patients. Additionally, geometric inconsistency in the predicted depth will be addressed, potentially by adopting a self-supervised network that includes both depth and pose prediction, which may simultaneously be a more end-to-end estimation network with improved efficiency. With estimated poses, we also intend to explore the possibility to combine EM-acquired poses and poses estimated from monocular images such that stability of a surgical AR system could be improved.

## Footnotes

1 https://www.iris.mech.hku.hk/surgical-planning.

2 https://youtu.be/kCi1ux-Q1FQ.
